# Late-onset pulmonary benign metastasizing leiomyoma 3 decades after hysterectomy: A case report

**DOI:** 10.1016/j.radcr.2025.12.008

**Published:** 2026-01-07

**Authors:** Christopher Kaleb Romero Ríos, Byron R. Larios Alemán, Andres S. Zamora

**Affiliations:** a School of Medicine, Hospital Militar Escuela “Dr. Alejandro Dávila Bolaños”, Managua, Nicaragua; b Department of Radiology, Hospital Militar Escuela “Dr. Alejandro Dávila Bolaños”, Managua, Nicaragua

**Keywords:** Abdominopelvic imaging, Pulmonary nodules, Uterine fibroid, Late metastasis, Benign metastasizing leiomyoma

## Abstract

Uterine leiomyomas are the most common benign tumors of the female reproductive tract. In rare cases, they may disseminate beyond the pelvis without malignant features, a condition known as benign metastasizing leiomyoma (BML). The lungs are the most frequent site of involvement, typically appearing years after hysterectomy. We report a 74-year-old woman with a history of hysterectomy at age 43 who underwent resection of a pelvic mass in 2022, which was diagnosed as a cellular leiomyoma. In 2025, surveillance imaging revealed a multilobulated pelvic mass and multiple bilateral solid pulmonary nodules, the largest measuring 9.5 mm, without mediastinal lymphadenopathy. The patient remained asymptomatic (ECOG 0). A multidisciplinary team opted for active surveillance, given the benign histology and the absence of clinical progression. This case highlights that benign metastasizing leiomyoma (BML) can present even several decades after uterine surgery, emphasizing the need to consider this diagnosis in women with a gynecological history who develop pulmonary nodules. It also underscores the central role of computed tomography in detecting both typical patterns of pulmonary dissemination and atypical findings such as potential intravascular extension. Finally, it illustrates that despite their benign histology, cellular leiomyomas may display unpredictable biological behavior, making multidisciplinary evaluation and careful radiological follow-up essential before pursuing more aggressive therapeutic approaches.

## Introduction

Uterine leiomyomas are the most common benign tumors of the female reproductive tract, affecting over 70% of women worldwide. These hormone-dependent tumors are characterized by the proliferation of uterine smooth muscle cells embedded within an abundant extracellular matrix. Although benign, uterine fibroids can cause significant morbidity, representing the primary indication for hysterectomy and constituting an important source of gynecological and reproductive dysfunction, including menorrhagia, pelvic pain, infertility, recurrent miscarriage, and preterm labor [1]. In rare cases, they may disseminate to extrapelvic sites without exhibiting malignant features, a condition known as benign metastasizing leiomyoma (BML). This phenomenon predominantly affects the lungs and is associated with hematogenous dissemination of uterine leiomyoma cells [2].

## Case presentation

A 74-year-old woman with a history of type 2 diabetes mellitus, arterial hypertension, and hypertensive heart disease with preserved ejection fraction was under oncological follow-up. In February 2022, she underwent resection of a pelvic mass. Given her history of hysterectomy at age 43 due to uterine fibroids, the mass was attributed to the proliferation of residual smooth muscle tissue in the pelvis.

**Fig. 1:**
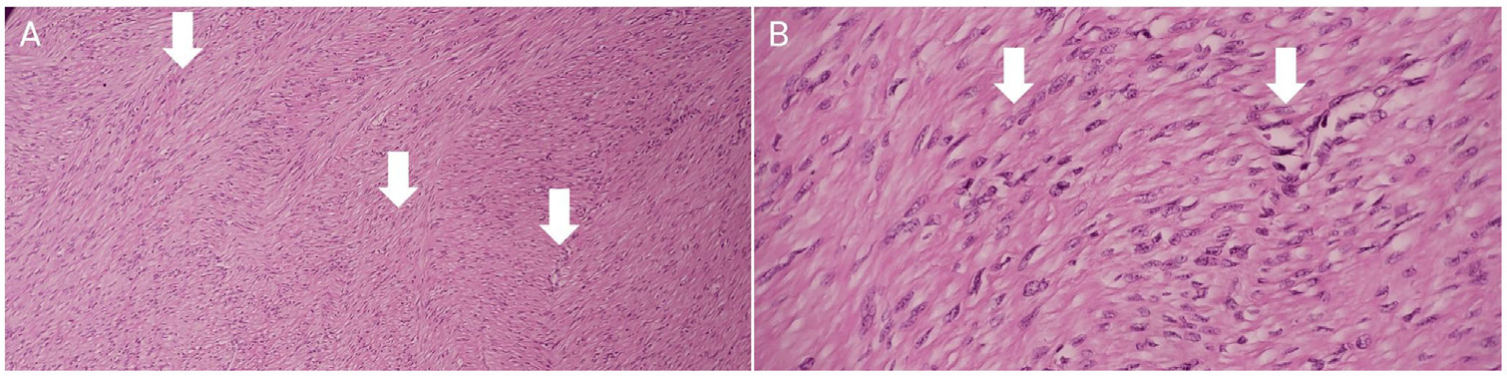
Histopathology stained with hematoxylin and eosin. A: 10X, B: 40X. Spindle-shaped cells with eosinophilic cytoplasm (white arrows) and indistinct cell borders, lacking cytological atypia and mitotic figures, arranged in intersecting fascicles.

Histopathological analysis revealed a benign mesenchymal neoplasm composed of a high proliferation of spindle cells arranged in a fascicular architectural pattern, with additional areas displaying a palisading arrangement. No nuclear atypia was identified, and mitotic activity was low. These cells showed an immunophenotypic profile of smooth muscle differentiation, expressing positivity for smooth muscle actin and desmin, which confirmed the diagnosis of cellular leiomyoma (Fig. 1).

**Fig. 2:**
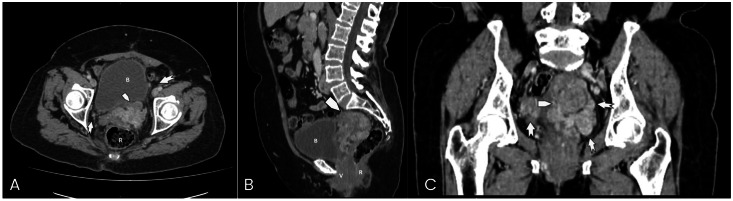
Uterine leiomyoma in a 74-year-old female patient. Contrast-enhanced venous phase abdominal CT. The images demonstrate a multilobulated pelvic mass (pentagon arrow) occupying the pelvic cavity, showing heterogeneous enhancement and loss of normal morphology due to multiple diffuse nodular lesions, some of them exophytic, with post-contrast enhancement. Adjacent pelvic lymph nodes (dentate arrow) with preserved morphology are also seen, all measuring less than 15 mm. A: axial view; B: sagittal view; C: coronal view. R: rectum; B: bladder; V: vagina.

**Fig. 3:**
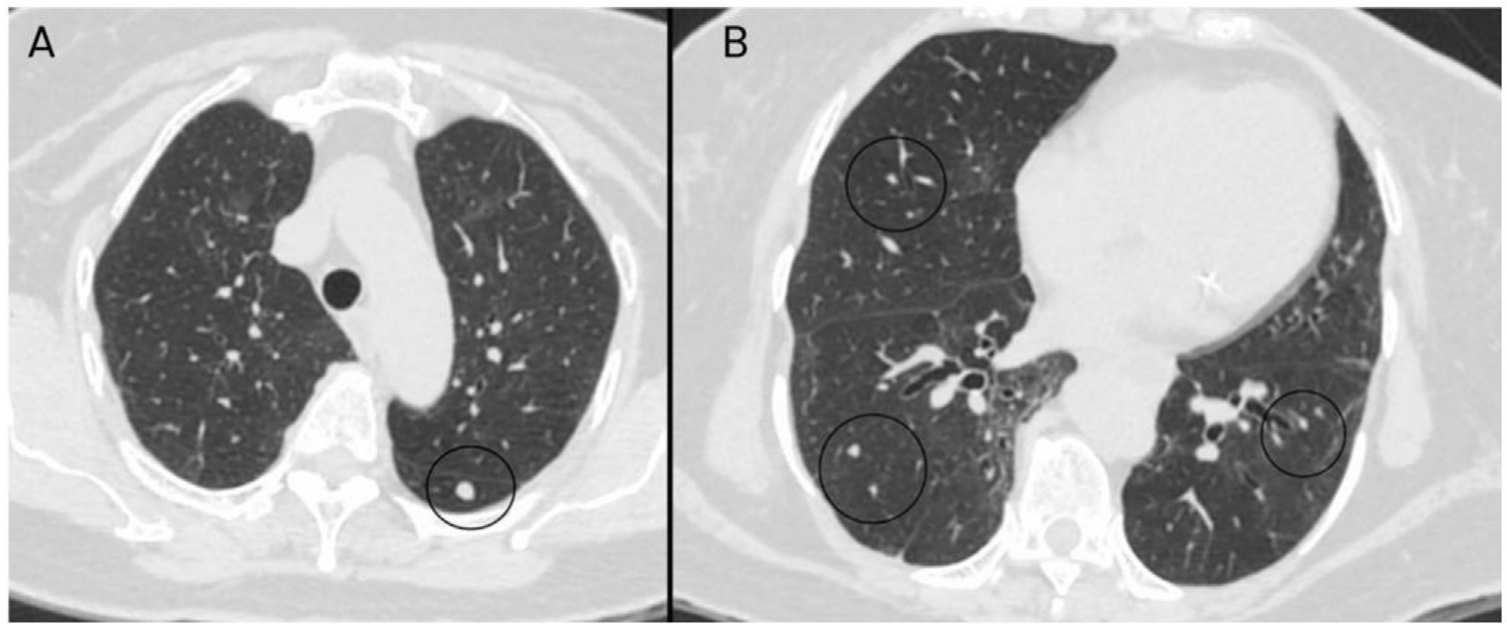
Chest CT scan performed for staging purposes. In lung window: multiple solid nodules (black circle) with well-defined margins are identified, distributed in isolation across both lung fields. These nodules measured between 5 and 9 mm. Additional findings include laminar atelectasis. A: Axial view at the apices. B: Axial view at the level of T9.

**Fig. 4:**
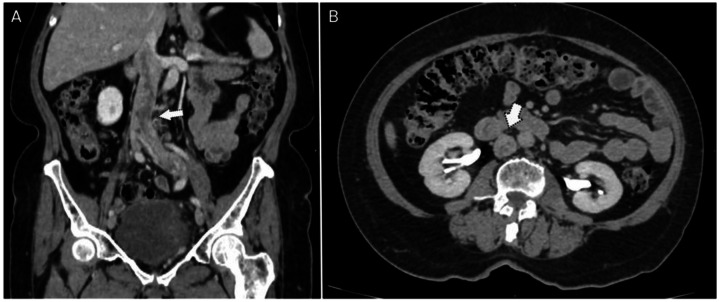
Contrast-enhanced abdominal CT Venous phase (A, B) and delayed phase (C). Filling defect in the left common iliac vein with extension into the inferior vena cava. The left common iliac vein is enlarged compared with the contralateral side, consistent with consistent with thrombosis or intravascular tumor extension (dotted arrow). A: Coronal view. B: Sagittal view. C: Axial view at the infrarenal portio.

For more than 2 years after surgery, her clinical course remained stable without signs of tumor activity. However, during routine oncologic surveillance in March 2025, a contrast-enhanced computed tomography (CT) scan of the abdomen and chest was performed (Fig. 2, Fig. 3, Fig. 4). The abdominopelvic study demonstrated a multilobulated pelvic mass in close contact with the rectum, bladder, and pelvic walls, displacing adjacent structures without overt infiltration. The lesion measured approximately 73 × 54 × 58 mm, with an estimated volume of 119 cc. Additionally, a partial filling defect was observed in the left iliac vein and inferior vena cava, suggestive of thrombosis or, in the context of known neoplasia, possible intravascular tumor extension.

The most significant thoracic finding was the presence of multiple solid pulmonary nodules with bilateral, centrilobular distribution. The largest, located in the superior segment of the left lower lobe, measured 6.5 mm; another in the right lower lobe measured 4.5 mm, with additional smaller nodules in the left upper and right middle lobes. No mediastinal or hilar lymphadenopathy was noted. Other findings included cylindrical bronchiectasis at the lung bases and a sliding hiatal hernia.

The patient was asymptomatic from a respiratory perspective and functionally independent (ECOG 0). She was referred to medical oncology to determine the most appropriate management, considering options such as active surveillance versus systemic therapy. Given the imaging characteristics and the patient’s clinical stability, the differential diagnosis included BML versus a low-grade neoplasm with limited metastatic potential. Gynecologic oncology recommended expectant management in coordination with clinical oncology, with follow-up scheduled in 3 months.

## Discussion

Uterine fibroids, also known as leiomyomas, are the most common benign neoplasms of the female reproductive tract, affecting over 70% of women by the age of 50 and reaching prevalence rates exceeding 80% among Black women [3,4]. Histologically, they are composed of benign smooth muscle cells embedded in a collagen, fibronectin, and proteoglycan-rich matrix [5], and their development is influenced by genetic, hormonal, racial, and environmental factors [3]. While most are asymptomatic, a significant proportion may present with symptoms such as abnormal uterine bleeding, pelvic pain, or infertility.

On rare occasions, uterine leiomyomas can spread beyond the uterus, giving rise to a condition known as benign metastasizing leiomyomatosis (BML), with the lungs being the most common site of distant implantation [6,7]. Fewer than 200 cases have been reported in the literature to date [6], with a mean age at diagnosis of 47 years and a typical interval of 8 to 15 years between the initial uterine surgery and the appearance of pulmonary nodules [8]. In the present case, the patient developed pulmonary findings approximately 33 years after undergoing hysterectomy, which represents an unusually long interval compared to typical reports in the literature, where the average ranges from 8.8 to 14.9 years [8].

The most widely accepted theories regarding the pathophysiology of BML include hematogenous dissemination of uterine smooth muscle cells, supported by studies demonstrating identical clonal genomic patterns between primary uterine tumors and pulmonary nodules [9,10]. Additionally, specific genetic alterations, such as loss of chromosomes 22q, 3q, and 11q, have been proposed to define BML as a distinct biological subtype within the spectrum of uterine leiomyomas [11]. At the molecular level, recent studies have proposed the use of biomarkers such as miRNA-221 (overexpressed in more aggressive forms) and miRNA-126 (underexpressed in pulmonary BML) as potential predictors of tumor behavior and for differentiation between BML and low-grade leiomyosarcoma [12].

From an imaging standpoint, chest computed tomography (CT) is essential for detecting pulmonary nodules associated with BML. These nodules are typically multiple, bilateral, solid, well-defined, and smooth-bordered, without spiculation or signs of aggressiveness [13]. In this case, the patient presented with multiple solid centrilobular nodules with bilateral distribution, the largest measuring 6.5 mm, in the absence of mediastinal lymphadenopathy. These findings are consistent with those described by Horstmann et al. in their case series, in which 70% of patients had multiple well-defined nodules without lymph node involvement [14].

CT imaging also revealed a multilobulated pelvic mass and a partial filling defect in the left iliac vein and inferior vena cava. This finding raises the differential diagnosis between a bland thrombus and intravascular tumor extension. Differentiation is critical; while bland thrombi are typically non-enhancing, tumor thrombi often exhibit contrast enhancement and direct continuity with the primary pelvic mass [15]. In this patient, the defect was suggestive of tumor extension, a feature documented in aggressive variants such as intravenous leiomyomatosis [15].

Although definitive diagnosis requires histological confirmation, radiologic findings especially in the context of a history of treated uterine fibroids can strongly suggest BML. The absence of significant cellular atypia, low mitotic activity, and positivity for hormone receptors on biopsy usually confirm the benign nature of the process. In this case, pulmonary biopsy was not performed, as radiologic findings were compatible, and the patient remained asymptomatic with excellent functional status (ECOG 0).

Management depends on the patient’s clinical status and the progression of the lesions. A clear hormonal component has been recognized, as these lesions tend to be estrogen- and progesterone-dependent [16]. In asymptomatic cases with stable disease, periodic observation through imaging may be sufficient [[17], [18]]. For patients with symptoms or documented progression, first-line treatment includes GnRH agonists or hormonal inhibitors, with favorable responses reported in several case series [[18], [19]]. Pulmonary surgical resection is reserved for selected cases, particularly when diagnostic uncertainty exists or there is significant functional compromise [[19], [20]].

## Conclusions

This case illustrates an unusually delayed presentation of BML, with pulmonary nodules emerging 33 years after hysterectomy for uterine fibroids. Despite benign histology, cellular leiomyomas can exhibit unpredictable biological behavior, including distant spread, posing significant diagnostic challenges. CT imaging played a pivotal role in detecting the characteristic pattern of multiple, well-defined, bilateral pulmonary nodules in an asymptomatic patient, as well as possible intravascular tumor extension, guiding a non-invasive initial approach. This case underscores the importance of including BML in the differential diagnosis of pulmonary nodules in women with relevant gynecologic history even decades after uterine surgery and highlights the value of multidisciplinary evaluation and careful radiologic surveillance in atypical presentations.

## Patient consent

Written informed consent was obtained from the patient for the publication of this case report and any accompanying images. All personal identifiers have been removed or anonymized to protect the patient’s privacy and confidentiality.
